# Applying Machine Learning Approaches to Suicide Prediction Using Healthcare Data: Overview and Future Directions

**DOI:** 10.3389/fpsyt.2021.707916

**Published:** 2021-08-03

**Authors:** Edwin D. Boudreaux, Elke Rundensteiner, Feifan Liu, Bo Wang, Celine Larkin, Emmanuel Agu, Samiran Ghosh, Joshua Semeter, Gregory Simon, Rachel E. Davis-Martin

**Affiliations:** ^1^Departments of Emergency Medicine, Psychiatric, and Population and Quantitative Health Sciences, University of Massachusetts Medical School, Worcester, MA, United States; ^2^Data Science Program, Computer Science Department, Worcester Polytechnic Institute, Worcester, MA, United States; ^3^Department of Population and Quantitative Health Sciences and Radiology, University of Massachusetts Medical School, Worcester, MA, United States; ^4^Departments of Population and Quantitative Health Sciences and Emergency Medicine, University of Massachusetts Medical School, Worcester, MA, United States; ^5^Department of Emergency Medicine, University of Massachusetts Medical School, Worcester, MA, United States; ^6^Computer Science Department, Worcester Polytechnic Institute, Worcester, MA, United States; ^7^Department of Family Medicine and Public Health Sciences, Wayne State University School of Medicine, Detroit, MI, United States; ^8^Department of Electrical and Computer Engineering, Boston University, Boston, MA, United States; ^9^Kaiser Permanente Washington Health Research Institute, Seattle, WA, United States; ^10^Departments of Emergency Medicine, Family Medicine and Community Health, University of Massachusetts Medical School, Worcester, MA, United States

**Keywords:** suicide prevention, mental health, predictive analytics, machine learning, neural network analysis

## Abstract

**Objective:** Early identification of individuals who are at risk for suicide is crucial in supporting suicide prevention. Machine learning is emerging as a promising approach to support this objective. Machine learning is broadly defined as a set of mathematical models and computational algorithms designed to automatically learn complex patterns between predictors and outcomes from example data, without being explicitly programmed to do so. The model's performance continuously improves over time by learning from newly available data.

**Method:** This concept paper explores how machine learning approaches applied to healthcare data obtained from electronic health records, including billing and claims data, can advance our ability to accurately predict future suicidal behavior.

**Results:** We provide a general overview of machine learning concepts, summarize exemplar studies, describe continued challenges, and propose innovative research directions.

**Conclusion:** Machine learning has potential for improving estimation of suicide risk, yet important challenges and opportunities remain. Further research can focus on incorporating evolving methods for addressing data imbalances, understanding factors that affect generalizability across samples and healthcare systems, expanding the richness of the data, leveraging newer machine learning approaches, and developing automatic learning systems.

## Background

According to the World Health Organization, approximately 800,000 people die by suicide annually worldwide, making it the 18th leading cause of death (1)^1^. In the United States, 48,344 people died by suicide in 2018 (2)^2^, making it the tenth leading cause of death and contributing to decreasing average United States life expectancy (3). Ultimately, the first step of suicide prevention can be viewed as a classification task to accurately identify individuals at risk for suicide in a specified time horizon, thereby allowing preventive intervention. However, the largest meta-analysis of suicide prediction (4) analyzed 365 studies and concluded that predictions based on individual risk or protective factors have led to weak predictive accuracy showing little improvement over time.

Several factors contribute to this prediction failure. Most notably, suicide is an uncommon event, even among those considered at high risk, such as individuals who have been psychiatrically hospitalized, making it inherently difficult to predict. In addition, suicide results from a complex interaction of numerous factors, each having small but meaningful contributions, rather than a handful of powerful stable predictors. Complicating matters, many suicide drivers are time-varying. Some might change slowly, such as major depressive episodes, while others may change quickly, such as acute alcohol or other substance intoxication (5, 6) or feelings of rejection following a relationship breakup. Prior studies were often limited to small samples and examined a limited number of factors, measured at a single time point, and focused predominately on stable or enduring factors. Consequently, previous efforts have not collected sufficiently comprehensive chronic and transient risk factors over time within a sufficiently large sample to produce accurate prediction models.

Another limitation lies in traditional analysis of suicide data. Until recently, classical statistical approaches predominated, primarily focusing on inference, which includes estimation and hypothesis testing for model parameters. This approach yields relatively simple models, emphasizing interpretability over prediction accuracy, and is not well-suited to handle data with many correlated, interacting factors, or programmed to incorporate new data to iteratively update the models.

However, two recent developments have transformed the suicide prediction landscape. First, large, complex, longitudinal databases, often referred to as “big data,” have been developed. For instance, adoption of electronic health record (EHR) systems has become ubiquitous (7), leading to an exponential data expansion: an estimated 2,314 exabytes (exabyte = one billion gigabytes) have been produced through 2020 (8)^3^. EHR data contains both structured and unstructured (text) data from multiple sources, is longitudinal, and can be linked with other sources, such as vital statistics and census data. Access to large, rich datasets containing substantial numbers of suicide cases is making it possible to overcome low occurrence rates.

Second, flexible mathematical and statistical models, referred to collectively as machine learning, have emerged, showing promise in addressing many problems inherent in previous approaches. Machine learning is well-suited to capitalize on emerging big data and enhanced computer processing capacity, making it feasible, easier, and cheaper to run massive analyses (9).

## Methods

This paper provides an overview of machine learning applied to suicide prediction, summarizes exemplar published studies for illustration, and explores future directions for research. The exemplar studies were selected based on consensus of the study team. Team members nominated papers from highly regarded research teams published in high-impact journals with content that aligned strongly with the relevant machine learning principles reflected in this paper. Then, the team worked together to identify the specific ones with the best fit.

## Results

### Machine Learning Overview

This section provides a high-level overview of machine learning. The Supplement has more technical details, with Supplementary Table 1 providing commonly used machine learning terminology. While there is no universally accepted definition of machine learning, typically, a dataset is created that includes predictors, often referred to as attributes or features, along with corresponding known outcomes, often referred to as labels, creating what is referred to as a labeled dataset. This approach is called supervised learning. Then, a function (model) can be inferred (learned or trained) to map an input (a set of predictors) to the output (its corresponding label), taking into account the relevant interactions and relations among the predictors. The learning process is optimized such that the derived labels from the learned function can be as accurate as possible compared with correct labels, with good generalizability to unseen data. In suicide prediction, the attributes or features (predictors) for supervised learning would be a specific individual's characteristics, such as demographics, psychiatric diagnoses, substance abuse disorders, and emergency department utilization history. Their corresponding outcome would indicate whether the individual died by suicide (10). These labeled data are used to train a model, with the specific training process dependent on the machine learning algorithm employed (described further in section Common Supervised Learning Approaches).

Machine learning allows the data itself to drive discovery by exploiting patterns or associations in the data without making *a priori* assumptions about distributions or formulating specific hypotheses. Consequently, machine learning can synthesize complex data with a large number and rich variety of variables and interactions.

Machine learning explicitly seeks to address rarity of suicide, referred to as data imbalance. While increasing the training dataset size helps address imbalance by providing more suicide cases, massive datasets with millions of cases are typically infeasible, and, even when available, they still do not completely solve data imbalance. Fortunately, imbalance mitigation strategies have been developed and are evolving. One common strategy involves under-sampling the majority class, that is, those that did not die by suicide, and over-sampling the minority class, that is, those that died by suicide, to create more balanced datasets. More sophisticated sampling methods, such as synthesizing new variants of the existing cases (11), are also popular. Ensemble methods (12) can be utilized wherein multiple models use the same minority class cases while each model works with distinct subsets of majority class cases. These trained models are then ensembled into one final classifier that combines their respective predictions into a final prediction. Further, cost-sensitive learning (13) tackles imbalance by assigning higher misclassification costs with the minority class and seeks to minimize high cost errors. Lastly, as accuracy may not be a meaningful metric when applied to imbalanced data, performance metrics that explicitly account for such imbalance, such as F1-score, can be applied with unbalanced training datasets. The Supplementary Table 2 depicts the basic classification structure underlying most approaches, and Supplementary Table 3 has definitions of common evaluation metrics.

Machine learning incorporates strategies for assuring robustness against overfitting (14), which is when a model is very specific to a training dataset but fails when applied to new datasets. Overfitting is more likely when the model is excessively complex, or when the number of variables or features is very large, but the data size is small. Applied to suicide prediction, overfitting might occur when too many predictive factors such as demographics, risk factors, stressors, and symptom inventories are used. Some strategies for protecting against overfitting include regularization (artificially enforcing smoothness in the model), early stopping (stopping iterations when a particular performance level is reached), or ensembling (combining predictions from multiple independent models). However, overfitting can nevertheless arise. Transfer or replication of a model trained on one dataset to other datasets derived from a similar target population is required. Only after rigorous cross-sample replication and attention to the parameters that might influence generalizability can we be confident the model is robust, valid, and ready for clinical translation.

Machine learning models can adapt over time. This means they can learn directly from data fed back into the model over time without requiring explicit human instruction. This is desirable for tasks too complex for complete manual enumeration of all precise rules or not completely understood by humans. For instance, increasingly larger combinations of newly discovered risk factors as well as the relationship between them might need to be taken into account for achieving effective suicide prediction. It would not be feasible to manually derive the complete set of all valid logic rules to fully capture the true relationship between predictors and suicide (15)^4^. Instead, the model can be designed to adapt over time based on prediction accuracy and as new variables are introduced and new data entered into the model.

#### Common Supervised Learning Approaches

While many supervised learning approaches can be applied to classification and prediction, four are widely used and have been applied to suicide: penalized regressions, decision trees, random forests, and Support Vector Machines (SVMs) (9). Supplementary Table 4 provides a summary of strengths and weaknesses of these approaches, while Supplementary Figure 1 provides an example of a supervised learning algorithm.

Penalized regression refers to a class of iterative methods that determine optimal regression coefficients subject to certain constraints to avoid overfitting. Variants that have been explored for suicide prediction include Lasso (16) and Elastic Net (17). These variants differ primarily in the constraints applied, such as reducing the weight of the coefficients of certain features vs. completely eliminating them by setting them to zero.

A decision tree learns in a hierarchical fashion by iteratively splitting the dataset into increasingly smaller subsets based on decision criteria on a given variable. The construction of this decision tree aims to produce the most homogeneous group possible at each split (18, 19). An example of an initial split might be whether the individual has a well-known risk factor, such as bipolar disorder, and a second split might involve a second factor, such as access to a firearm. Random forest, an extension of decision trees, uses majority voting to combine decisions from multiple decision tree models that are created from different subsets of the same dataset in order to produce a final classification decision.

Another widely used supervised learning algorithm is an SVM, which belongs to the class of methods that jointly performs classification in a single decision step. SVM aims to find a decision boundary, called a hyperplane, that best divides different classes (e.g., suicide vs. not suicide) in high-dimensional space (i.e., a large number of possible predictors). The optimal hyperplane is computed using the “max-margin principle,” such that data instances that are the nearest to the hyperplane, but from different classes (called the support vectors), are separated by the widest possible margin. New data examples are then mapped into that same space and predicted to belong to a class label based on the side of the hyperplane on which they land. SVMs are versatile in that they can handle sparse data and are widely applicable to numeric data.

Typically, multiple approaches are used on the same data and accuracy compared to select the best performer. Besides accuracy, interpretability of the results can be used to decide the best approach. Unlike regressions and decision trees, SVM results are not easily interpretable due to mapping of data instances into an abstract representational space and employing vectors as decision boundaries in this space. In situations where interpretability is important, regressions and decision trees are preferred.

#### Leveraging Unstructured Data Through Natural Language Processing

All machine learning approaches are applicable to structured data; they assume numeric or categorical data as input. However, much EHR data consists of unstructured narrative notes. Natural language processing (NLP) strategies have been developed to process human language by applying syntactic and semantic knowledge and extracting structured concepts that can serve as features characterizing the patient. For example, text that describes firearm ownership can be translated into binary values “yes” or “no.” With clinical narratives becoming increasingly available in health system databases, NLP has become an essential tool for constructing clinically relevant structured information (20, 21). Providing early support, McCoy et al. (20) used off-the-shelf NLP technology to develop algorithms from narrative discharge summaries that better predicted suicide and accidental death after hospital discharge than traditional structured data alone.

### Case Studies on Estimating Risk for Suicide

A recent meta-analysis concluded that emerging machine learning studies have led to significantly better prediction of suicide-related outcomes than earlier studies using smaller samples and classical statistical approaches (22). This literature will not be reviewed in depth, but, instead, two exemplar studies that predicted death by suicide will be used for illustration purposes.

Simon et al. used Mental Health Research Network data containing historic EHR structured data from seven civilian health systems linked with death data (16). The sample consisted of 2,960,929 individual patients ≥13 years old who contributed 19,961,059 eligible primary care or outpatient specialty mental health visits between January 1, 2009 and June 30, 2015. In the 90 days after an eligible visit, 24,133 suicide attempts and 1,240 suicide deaths were identified. The investigators developed a logistic regression model with penalized Lasso variable selection, described in section Common Supervised Learning Approaches, to predict suicide deaths and suicide attempts. Input variables (features), spanning up to 5 years before the index visit, included socio-demographic variables (e.g., age, sex, neighborhood income), current and past mental health and substance abuse diagnoses, past suicide attempts, past injury or poisoning, in-patient and emergency service use, psychotropic medications, general medical morbidity measured by Charlson Comorbidity Index (23)^5^ categories, and the Patient Health Questionnaire −9 (24), a patient-reported depression severity measure. Importantly, they factored in time windows for diagnoses and acute care utilization to represent within 90 days, 1 year, and 5 years of the index visit, as well as numerous interactions between socio-demographics and health care features.

The final input pool comprised 313 variables. Suicide was rare, occurring in <1 tenth of 1% of the sample. Despite this, the model predicting suicide in 90-days after a visit had a c-statistic, synonymous with a receiver operating characteristic curve's area under the curve, of 83%-86%. Visits with risk scores above the 75th percentile identified 80% of subsequent suicide, while those above the 95th percentile identified 43% of all suicide. This accuracy was markedly greater than previously published efforts (25, 26) and was superior to several widely used medical outcome prediction tools, such as predicting rehospitalization for heart failure (27) and in-hospital mortality from sepsis (28). These historic results were likely due to several factors, including a very large dataset, enhanced ascertainment of risk factors present in EHRs, using a very large predictor pool, including interaction terms, use of temporal coding, use of robust machine learning analytic strategies, and inclusion of a patient reported measure, the Patient Health Questionnaire-9, which accounted for significant prediction variance despite being available for <20% of the sample.

A similar study completed by Kessler and colleagues (17) used EHR data from United States Veterans Health Administration. Their sample included 6,359 veterans who died by suicide during 2009–2011 and used Veterans Health Administration services in the year of their death or the prior year and a randomly selected control sample of 2,108,496 veterans who received Veterans Health Administration services but were alive at the end of the month the suicide decedent died. They began with 381 predictors spanning several domains similar to the Mental Health Research Network study, except Patient Health Questionnaire-9 scores were not available. They also incorporated time varying predictors for healthcare utilization and mental health diagnoses spanning 1, 2, 3, 6, 12, 19, and 24 months prior to the index visit. Their primary analysis used a penalized logistic regression Lasso approach to predict suicide within a 30-day window and found very promising results. Their final algorithm, which used 61 of the original 381 predictors, revealed sensitivities among individuals with the top 0.1, 1, and 5% of risk as 2.8, 11.8, and 28.2%, respectively. These sensitivities are low in absolute terms but are markedly better than historical efforts (4). Also, they were replicated when applied to an independent prospective validation sample.

Notably, Kessler's study also evaluated eight additional machine learning approaches that allow complex non-linear interactions among predictors, including those that can maximize prediction accuracy but are uninterpretable “black box” approaches, like SVM. These algorithms revealed similar sensitivities as the Lasso regression, and, one approach, a version of decision tree analysis, showed slightly stronger prediction accuracy. The authors encouraged validation with other datasets before their decision tree findings can be interpreted as reliably and meaningfully superior.

## Discussion

Initial efforts using machine learning to predict suicide are promising; however, the field remains in its early stages and much work remains before these approaches can be fully embraced clinically. Below, directions for future research are discussed.

### Incorporating Time

Future machine learning efforts will need to address time from several perspectives. First, historical factors preceding the prediction point (the visit or date when the estimation of future probability is being made) require time boundaries, because the nearness of the feature itself may have differential associations with future event probability. A suicide attempt in the month before the prediction point may be more strongly associated with an attempt in the next 3 months than a suicide attempt 30 years ago. Second, the time horizon, or prediction window, after the prediction point is important; different features may predict short-term suicide compared to long-term suicide. Third, for features expected to fluctuate quickly, such as mood states, frequent assessment and longitudinal representation in datasets are ideal. Fourth, time, as embodied by an individual's age, likely influences model composition; suicide drivers among adolescents may be very different than drivers among the elderly. Careful attention to the variety of time-related issues are essential for building models that can adjust an individual's estimated risk based on age, modified as time passes, and trigger interventions tailored to short- vs. long-term risk.

In addition to incorporating time into model construction, future studies need to build learning models that digest new data, new predictors, suicide outcomes, and timely human feedback, leading to a continuous learning loop that improves prediction performance iteratively over time. This is a fundamental advantage of machine learning yet, to date, all published machine learning suicide studies report on static models developed using an initial database within a given time window. Building automated learning models would empower us to fully realize the value of machine learning.

### Incorporating New EHR Features and Data Sources

A model is only as good as the richness of the data input, meaning classification (prediction) accuracy for future studies will benefit from new and evolving features as they become available in EHRs, such as data obtained from suicide-specific risk screening and assessments. These instruments are increasingly being adopted by health systems because of organizations such as the Joint Commission (29)^6^ that are promoting new standards, which in turn are being built as EHR templates. Moreover, the use of standardized measures to guide care decisions, or measurement-based care, is becoming more common (30, 31). This means serial administration of patient reported measures, like the Patient Health Questionnaire-9, and integration of even more sophisticated measures, like computerized adaptive tests (32, 33), into EHRs are likely to improve our ability to accurately measure time-varying features, like psychiatric symptoms, escalations in substance use, and stress. Other data sources outside the EHR may be linked to improve data richness, such as small area geocode variables, judicial and penal system records, biomarkers and genomics, social media data, and mobile application data. Linking these rich data sources would mitigate weaknesses inherent in current EHR datasets by enabling a more comprehensive and time-sensitive set of inputs, which may improve accuracy while helping adjust risk estimations over time.

### Applying Novel Advances in Machine Learning

Ensemble learning, which combines predictions from a variety of approaches rather than using just one, tends to offer better prediction than single strategies (34). Future studies will need to evaluate whether the ensemble approach is worth the increased complexity and cost. Further, an advanced type of machine learning, called deep learning (35),^7^ has shown promise in solving increasingly complex problems in other fields, such as recognizing objects inside of an image and understanding speech, movement, activity, sleep, and online behavior. Deep learning works by composing multi-layered non-linear computational neural network models inspired by the neural structure of the human brain. Because deep neural network models typically rely on an enormous number of labeled data instances, to fully utilize these strategies we will need to build even larger databases.

In addition to advances in computational approaches, NLP advances have created sophisticated strategies for translating text into meaningful structured data. For instance, bidirectional encoder representations from transformers (36) is a recent NLP neural language model developed by Google AI in 2018 and has demonstrated state-of-the-art results on a variety of NLP tasks. Bidirectional encoder representations from transformers contains a multi-layer neural network architecture (37)^8^ that can learn optimal vector representations of each word incorporating contextual information bidirectionally. The semantic-rich representations derived from EHR narratives through bidirectional encoder representations from transformers would likely further strengthen prediction models.

### Understanding Implementation

Just because suicide can be predicted successfully using a particular training dataset data does not mean these algorithms easily transfer into clinical practice. Understanding how well the algorithms translate from training data to an individual health system is essential. It is unlikely that a published algorithm can simply be “copied and pasted” into a health system. Before algorithms can be transferred to a given setting, we need to know more about factors affecting their accuracy, and, even then, a process for local validation using a health system's own data is likely necessary before algorithms can be translated into practice.

Future studies will need to evaluate the best way to visualize and communicate the results from these algorithms in such a way that they are intuitive, useful, and actionable for the clinician and the patient (38). The blending of machine learning prediction with clinician-based suicide risk assessments, what can be referred to as a “human in the loop” approach, needs exploration to develop effective decision support tools clinicians will trust and utilize. We need to better understand the relative value of each data source for its predictive power, and to drill down to understand when information sources contradict one another, such as when a machine learning algorithm suggests a person is at high risk but the individual denies thoughts of suicide.

An essential consideration for translation into practice is EHR alert fatigue. Because prediction is driven by many long-standing factors conveying long-term risk, such as the diagnosis of major depressive disorder, an EHR alert built on an algorithm tapping into this historical data will likely persist, even after treatment has been delivered or symptoms subside. Consequently, the alerts have the potential for diminishing utility over time. In some cases, this can have serious implications for patient outcomes. For example, if a clinician becomes fatigued and ignores these alerts, he or she may miss an opportunity to intervene with a suicidal patient. This is a practical example of why building models that adjust with time, both backward by incorporating time-bounded predictors and forward by incorporating specific time horizons, are essential. Static alerts that do not reflect changes in clinical status or interventions will quickly become ignorable.

## Conclusion

Machine learning has strong potential for improving estimation of future suicide risk and for monitoring changes in this risk over time; however, important challenges remain before this benefit can be realized clinically. Further research must address persistent methodological issues by incorporating novel methods for addressing data imbalance and overfitting and understanding factors that affect generalizability across samples and settings. Expanding the richness of the input data, leveraging newer analytic approaches, and developing automatic learning systems offer strong promise for both improving predictive ability and adjusting risk estimations over time. As important as pure predictive ability, we need to explore the best ways to represent risk to the clinician, so it is easily interpretable, actionable, and minimizes alert fatigue.

## Data Availability Statement

The original contributions presented in the study are included in the article/Supplementary Material, further inquiries can be directed to the corresponding author/s.

## Author Contributions

EB was responsible for the overall manuscript preparation, including overseeing and synthesizing edits from the rest of the authors. EB, ER, FL, BW, CL, EA, SG, JS, GS, RD-M contributed materially to the conceptualization, writing, and editing of this paper. All authors contributed to the article and approved the submitted version.

## Author Disclaimer

The content is solely the responsibility of the authors and does not necessarily represent the official views of the NIH.

## Conflict of Interest

The authors declare that the research was conducted in the absence of any commercial or financial relationships that could be construed as a potential conflict of interest.

## Publisher's Note

All claims expressed in this article are solely those of the authors and do not necessarily represent those of their affiliated organizations, or those of the publisher, the editors and the reviewers. Any product that may be evaluated in this article, or claim that may be made by its manufacturer, is not guaranteed or endorsed by the publisher.

## Footnotes

^1^ https://www.who.int/mental_health/prevention/suicide/suicideprevent/en/

^2^ https://webappa.cdc.gov/sasweb/ncipc/leadcause.html

^3^ https://med.stanford.edu/content/dam/sm/sm-news/documents/StanfordMedicineHealthTrendsWhitePaper2017.pdf

^4^ https://cdn.preterhuman.net/texts/science_and_technology/artificial_intelligence/Introduction%20to%20Machine%20Learning%20-%20Nils%20J%20Nilsson.pdf

^5^ https://healthcaredelivery.cancer.gov/seermedicare/considerations/comorbidity.html

^6^ https://www.jointcommission.org/resources/patient-safety-topics/suicide-prevention/

^7^ https://papers.nips.cc/paper/2016/file/577ef1154f3240ad5b9b413aa7346a1e-Paper.pdf

^8^ https://arxiv.org/pdf/1706.03762.pdf

## Abbreviations

EHR: electronic health record
SVM: support vector machines
NLP: natural language processing.
