# Supplementary material for: Applying Machine Learning Approaches to Suicide Prediction Using Healthcare Data: Overview and Future Directions
Source: Front Psychiatry. 2021 Aug 3;12:707916. doi: 10.3389/fpsyt.2021.707916 (PMC8369059; doi:10.3389/fpsyt.2021.707916)
Supplement: Supplementary file 1 [file Table_1.DOCX]

Table 1 provides a basic overview of machine learning terminology. It presents terms from the machine learning perspective, a description of each term, synonyms for the term, and an example from suicide research.

**Table 1. Basic machine learning terminology**

| **ML Terminology** | **Description** | **Synonyms or Similar Concepts** | **Symbol Notation** | **Example From Suicide Research** |
| --- | --- | --- | --- | --- |
| Training instance | An example input-output pair from training data, e.g. {*x_i_*,*y_i_*} is the *i*th pair | inference sample | {*x_i_,y_i_*} | *Each patient in the first “training” dataset* |
| Test instance | An example input from testing data to test the derived function | validation sample | *x_i_*$'$ | *Each patient in the second “testing” dataset* |
| Feature | A predictor, risk factor, or an independent variable, e.g. *x_ik_* is the value of the *k*th variable from the $i$th training instance | independent variable, indicator, predictor | *x_ik_* | *Risk factors like age category, gender, or attempt history* |
| Feature vector | Each element in the vector represents a feature, and the feature vector of a training instance is an input of the target function | sample profile, sample characteristics | *x_i_=(x_i1_,x_i2_,…,x_im_)* | *The vector [25, 0, 1] can represent a patient who is 25 years old (“25”), no smoking (“0”), and diagnosed as bipolar disorder (“1”)* |
| Classification label | The target variable or dependent variable, e.g. *y_i_* is the target variable of the *i*th training instance | dependent variable, target variable, healthcare outcome | *y_i_* | *Death by suicide* |
| True function | A function to map the feature vector of a training instance to its label | true population model | *f* | *Function that describes the actual relationship between risk factors and completed suicide for a given patient in the “training” dataset* |
| Target function | A function learned from training data to optimally approximate the true function | inferred sample model | *h* | *Function that attempts to predict whether completed suicide occurs in a new patient in the “testing” dataset* |
