# Supplementary material for: Applying Machine Learning Approaches to Suicide Prediction Using Healthcare Data: Overview and Future Directions
Source: Front Psychiatry. 2021 Aug 3;12:707916. doi: 10.3389/fpsyt.2021.707916 (PMC8369059; doi:10.3389/fpsyt.2021.707916)
Supplement: Supplementary file 2 [file Table_2.DOCX]

Evaluation metrics of a classification system are typically defined using elements from a 2x2 matrix in Table 2. For suicide prediction or classification, the target variable represents any suicide-related outcomes, including positive (e.g. suicide ideation) or negative (e.g. non-suicide ideation) outcomes. There are four possible cases when comparing the system derived outcome with the true outcome, as illustrated in Table 2.

**Table 2. Counting statistics for evaluating classification (prediction) systems**

|  | | True Outcome | |
| --- | --- | --- | --- |
|  |  | Positive | Negative |
| System Derived Outcome | Positive | True Positive (TP) | False Positive (FP) |
|  | Negative | False Negative (FN) | True Negative (TN) |
