# Supplementary material for: Applying Machine Learning Approaches to Suicide Prediction Using Healthcare Data: Overview and Future Directions
Source: Front Psychiatry. 2021 Aug 3;12:707916. doi: 10.3389/fpsyt.2021.707916 (PMC8369059; doi:10.3389/fpsyt.2021.707916)
Supplement: Supplementary file 3 [file Table_3.DOCX]

Table 3 provides a summary of commonly used metrics to evaluate a machine learning-based classification system. All of them are calculated using the elements defined in Table 3.

**Table 3. Summary of evaluation metrics of machine learning-based classification system**

| Metrics | Other Names | Description | Math Notation |
| --- | --- | --- | --- |
| Sensitivity | True positive rate; recall | Percentage of patients with a known suicidal ideation/behavior who can be correctly detected as positive by the classification system | TP/(TP+FN) |
| Specificity | True negative rate | Percentage of patients without a known suicidal ideation/behavior who can be correctly detected as negative by the classification system | TN/(TN+FP) |
| Positive predictive value | Precision | Percentage of patients being detected positive by the classification system truly have suicidal ideation/behavior | TP/(TP+FP) |
| Negative predictive value | n/a | Percentage of patients being detected negative by the classification system truly have no suicidal ideation/behavior | TN/(TN+FN) |
| False negative rate | n/a | Percentages of patients with a known suicidal ideation/behavior are wrongly detected by the classification system as negative | 1-Sensitivity; FN/(TP+FN) |
| False omission rate | n/a | Percentage of patients detected by the classification system as negative actually have suicidal ideation/behavior | 1- negative predictive value; FN/(TN+FN) |
| F1 score | n/a | Harmonic mean of precision and recall | 2*precision*recall/(precision+recall) |
| AUC | ROC_AUC; AU_ROC | Area under the receiver operating characteristic curve, measuring the overall ability of distinguishing between positive and negative cases considering various thresholds | area(ROC) |
