# Supplementary material for: Applying Machine Learning Approaches to Suicide Prediction Using Healthcare Data: Overview and Future Directions
Source: Front Psychiatry. 2021 Aug 3;12:707916. doi: 10.3389/fpsyt.2021.707916 (PMC8369059; doi:10.3389/fpsyt.2021.707916)
Supplement: Supplementary file 4 [file Table_4.DOCX]

Table 4 summarizes the relative strengths and weaknesses of mainstream supervised learning approaches for classification tasks.

**Table 4. Strengths and weaknesses of five mainstream machine learning classification algorithms**

| **Algorithms** | **Strengths** | **Weaknesses** |
| --- | --- | --- |
| Logistic Regression | probabilistic interpretation; provides statistical significance of features | underperforms for non-linear decision boundaries; difficult to capture complex relationships |
| Decision Trees | robust to outliers; can model non-linear decision boundaries; easy to interpret; no need for feature normalization | prone to overfitting |
| Support Vector Machines | can model non-linear decision boundaries; robust against overfitting and outliers | memory intensive; doesn’t scale well to large data with large number of features; tricky to choose appropriate kernels; more complex to interpret for non-linear models |
| Random Forests | collective decision through ensemble; outperforms for many classification tasks; robust against overfitting | tricky to choose the number of trees; more complex to interpret than single decision tree |
| Deep Learning | outperforms for many classification tasks; avoid manual feature engineering; more expressive | typically needs large-size data to train; difficult to tune hyperparameters; computationally expensive; more complex to interpret |
