# Supplementary material for: Applying Machine Learning Approaches to Suicide Prediction Using Healthcare Data: Overview and Future Directions
Source: Front Psychiatry. 2021 Aug 3;12:707916. doi: 10.3389/fpsyt.2021.707916 (PMC8369059; doi:10.3389/fpsyt.2021.707916)
Supplement: Supplementary file 5 [file Table_5.DOCX]

Figure 1 below shows a learning algorithm with *n* training instances, each being represented by *m* variables. The algorithm tries to find an optimal function *h* from a class of functions *H* such that *h* can approximate the true function *f* that maps each training instance (represented by a feature vector) to its label, i.e. *Y*=*f*(*X*). Table 1 defines some terminology. In the context of applying machine learning to suicide, each patient in the first, “training” dataset is a “training instance”, various risk factors and clinical phenomenon extracted from this patient’s data are “features”, and the suicide outcomes of interest would be “classification labels.” The goal is to learn a function that would map each patient in the training data to different known suicidal outcomes, so that the function can be generalized to new patients (“test instances”) from the second dataset, the “testing” data, and hence predict their likelihood of a given suicidal outcome.

Figure 1. Demonstration of a supervised learning paradigm for classification [1]

**Supplement References:**

[1] Murphy KP. Machine learning: A probabilistic perspective. Cambridge, MA: MIT Press; 2012.
